# Probabilistic analysis and simulation of crack propagation in concrete pavements and surfaces

**DOI:** 10.1038/s41598-022-18060-8

**Published:** 2022-08-19

**Authors:** Moussa Leblouba, Mohamad Tarabin, Mostafa Zahri

**Affiliations:** 1 Department of Civil and Environmental Engineering, University of Sharjah, Sharjah, United Arab Emirates; 2 Department of Mathematics, University of Sharjah, Sharjah, United Arab Emirates

**Keywords:** Engineering, Materials science, Mathematics and computing

## Abstract

The surface of concrete pavement is susceptible to cracking. The propagation of a crack in a concrete structure may take the form of a one-dimensional line crack, a two-dimensional surface crack, or a three-dimensional volume crack. Predicting crack propagation in a concrete pavement surface is a complicated task. The whole process of formation, propagation, and orientation of cracks can be considered a stochastic process that necessitates a probabilistic investigation approach. In the present study, crack propagation in concrete pavements is studied from a probabilistic point of view using the concepts of multinomial Markov Chains using Random Walks. The study is based on actual probabilities of propagation and orientation of cracks obtained by tracing crack paths from a large dataset of images using custom-built software. Two random walker models are developed using the trinomial and multinomial Markov Chains. The master equation is developed assuming the trinomial Markov Chain, which has been brought for further theoretical and numerical developments. Examples of inferences and numerical simulations are presented to showcase the potential uses and applications of the proposed probabilistic approach for the design and scheduling of inspection visits and maintenance/rehabilitation strategies of concrete pavements and surfaces.

## Introduction

Rigid pavements are typically constructed using concrete and can be reinforced with steel. Either way, the pavement surface is susceptible to cracking, and thus, many repairs are needed. This is because of the nature of the materials used. The first step to devise a plan to repair the damaged portion of the pavement is to determine the cause of cracking. The most common causes of concrete pavement cracking are traffic or poor workmanship. Other causes can include poor design, cracked mortar joints, and excess water. Cracks in concrete pavements propagate at a rate of 25 mm per year in regular service.

The main consequences of crack development in concrete pavements are reduced pavement life, increased pavement maintenance costs, and increased traffic delays.

1. *Reductions in pavement life* Pavement life is reduced due to the increased stress and loading on the pavement caused by the cracks. This can lead to more frequent pavement rehabilitation or even replacement.
2. *Increased pavement maintenance costs* The cracks also create a condition that favors the ingress of water and other contaminants, leading to the corrosion of the reinforcing steel and the development of potholes and other pavement distresses. This requires more frequent and costly pavement maintenance.
3. *Increased traffic delays* The presence of cracks also creates a safety hazard, leading to the closure of lanes or even the entire roadway. This can lead to significant traffic delays.

The reason for concrete cracking is the presence of tensile stresses in the concrete due to the difference in the concrete compressive strength and tensile strength of aggregates. In new concrete, tensile stresses can be present due to the segregation of aggregates. Cracking can be observed in the form of spalling, delamination, surface cracking, and splitting of the concrete structure. Surface cracking is the most common form of cracking in concrete structures. Generally, these cracks propagate by a combination of two mechanisms^1,2^: (1) thermal expansion and contraction, which is the most common cause of concrete cracks and is due to the significant temperature variations that occur in concrete; (2) chemical reactions between the cement and water that form calcium hydroxide (Ca(OH)_2_) and portlandite (Ca(OH)_2_·5H_2_O).

Due to the many uncertainties associated with the concrete material constituents and loading conditions, a probabilistic approach is usually the only way to study crack propagation in concrete structures. A random walk model can be used to simulate the formation of cracks in a material^3^. In this model, a particle is randomly moved in one of two directions: left or right. If the particle moves left, it remains in the same position, and if it moves right, it advances one position to the right. If the particle moves off the edge of the material, it is removed from the simulation. This process is repeated for a set number of steps, and the position of any cracks that form is recorded. Additionally, the width of any cracks that form can be recorded and used to calculate the fracture toughness of the material.

A simple way to do this is to use a random number generator to create a series of random numbers between 0 and 1. For each number, determine whether it is less than or greater than 0.5. If it is less than 0.5, then the crack will move to the left. If it is greater than 0.5, then the crack will move to the right.

Other studies looked at the number of cracks in concrete slabs. Some^4,5^ found that the number follows a Poisson distribution.

## Crack propagation and orientation in concrete pavements

The propagation of a crack in a concrete structure may take the form of a one-dimensional line crack, a two-dimensional surface crack, or a three-dimensional volume crack. One-dimensional line cracks propagate straight, while two-dimensional surface cracks propagate in a curved or zigzag pattern. Three-dimensional volume cracks propagate in all directions. The rate at which a crack propagates depends on the properties of the concrete and the loading conditions. Concrete is a brittle material, and as a result, cracks tend to propagate quickly under tensile loading.

The prediction of crack propagation is a difficult task. In general, the crack propagation rate depends on the material properties, geometry of the structure, stress state, fatigue, and environmental conditions.

Many empirical formulas have been developed to predict the rate of crack propagation. One of the most commonly used formulas is the Paris law^6,7^. The Paris law originally proposed to predict the growth rate of discrete cracks in metals^8^ states that the crack propagation rate is proportional to the product of the stress intensity factor and the crack length; however, it does not account for crack initiation. Bazant and Xu^9^ and Slowik et al.^10^ were the first to apply the Paris law to concrete. Other factors that can affect the rate of crack propagation include the presence of corrosion, defects in the material, and foreign particles in the material.

In concrete pavements. cracks can be oriented in a variety of ways. The main factors that affect the orientation of the cracks are the thickness of the pavement, the temperature differential between the top and bottom of the pavement, and the amount of traffic on the pavement. Pavements with a significant temperature differential between the top and bottom are more likely to have cracks oriented perpendicular to the traffic flow. Pavements with much traffic are more likely to have cracks that are oriented parallel to the traffic flow.

## Studies available in the literature

Xi and Bazǎnt^11^ used a Markov process model to study the random growth of cracks. For that, they used the R-curve to characterize the growth process. Based on that, they were able to develop a model that predicts the growth of a crack. The focus of the publication is on the development of a model that predicts the growth of a crack. Then, they used the model to simulate the growth of a crack in a material. The use of the R-curve is important because it allowed the authors to identify the transition from a stable to an unstable fracture process. This is necessary to develop a model that can predict the growth of a crack. The model that the authors develop is based on the idea that the growth of a crack is a random process. This means that the growth of a crack is not predictable and can vary from one instance to the next. This makes it difficult to develop a model that can accurately predict the growth of a crack. The authors noted that their model is limited to cracks that grow in a straight line. This means that the model cannot be used to predict the growth of a crack that curves. Additionally, the model does not consider the influence of other cracks on the growth of a crack. The authors found that their model can predict the growth of a crack. Additionally, the model can predict the growth of a crack in the presence of other cracks. This is an important finding because it shows that the model can be used to predict the growth of a crack in a real-world scenario. However, the model has some limitations and needs to be further validated.

Bogdanoff and Kozin^12^ created a model that calculates the cumulative damage of an object over time. In addition, they did this by using probability theory. This article is important because it helps us understand how cumulative damage works. In addition, it can help us to create models that predict the cumulative damage of objects over time. Some of the limitations of this article include the fact that it is based on probability theory. This theory can be difficult to apply in some situations. Another limitation of this article is that it is based on a single point in time. It does not take into account the fact that cumulative damage can change over time. For further details on stochastic models of crack propagation in brittle materials, the reader is referred to reference^13^.

A number of studies have been conducted on crack propagation and orientation in concrete pavements^14,15^. Fathalla et al*.*^16^ used the data assimilation technology of multiscale simulation and the pseudo-racking method to investigate the effect of crack orientation on the fatigue life of RC bridge decks. They looked at a wide range of crack orientations to see how they affected fatigue life. The authors found that the crack orientation is highly associated with the coupled flexure shear mode of failure. The findings of this study have important implications for the maintenance and safety of RC bridges.

## Research objectives

Building on the abovementioned studies, the main objectives of the present paper are to:

1. Review the mechanisms of crack initiation, propagation, and orientation in rigid concrete pavements
2. Develop a computer program to trace crack paths and apply it to a database of real images of cracks in concrete pavements.
3. Develop two probabilistic random walk models for the prediction of the probability of a crack taking a certain path or reaching a certain location after a specified time.
4. Develop the master equation for the trinomial Markov Chain and numerically simulate the probabilistic crack propagation considering different cases.
5. Present definitions and one theorem on the computation of the probability of failure.
6. Present detailed numerical simulations and inference scenarios.

## Data collection

### Image database

Unfortunately, there is no definitive database of cracks in concrete pavements. In the present study, the authors resorted to Mendeley Data to search and collect concrete pavement surface cracks that have been uploaded by researchers. Several datasets containing images of cracked concrete can be found on the Mendeley database. Note that the dataset used in this study is licensed under the Creative Commons Attribution-ShareAlike 4.0 International License.

The dataset used for this study is the “Concrete Crack Segmentation Dataset”, which was collected from cracked pavements located in the Middle East Technical University^17^. This dataset contains over 400 high-quality concrete crack images, along with their alpha maps, which make it especially useful for training algorithms to detect and segment cracks in concrete images. The clearest 100 pictures that had good quality and no blurry parts were selected for use in this study, as that amount was determined to be sufficient to make conclusions.

**Figure 1:**
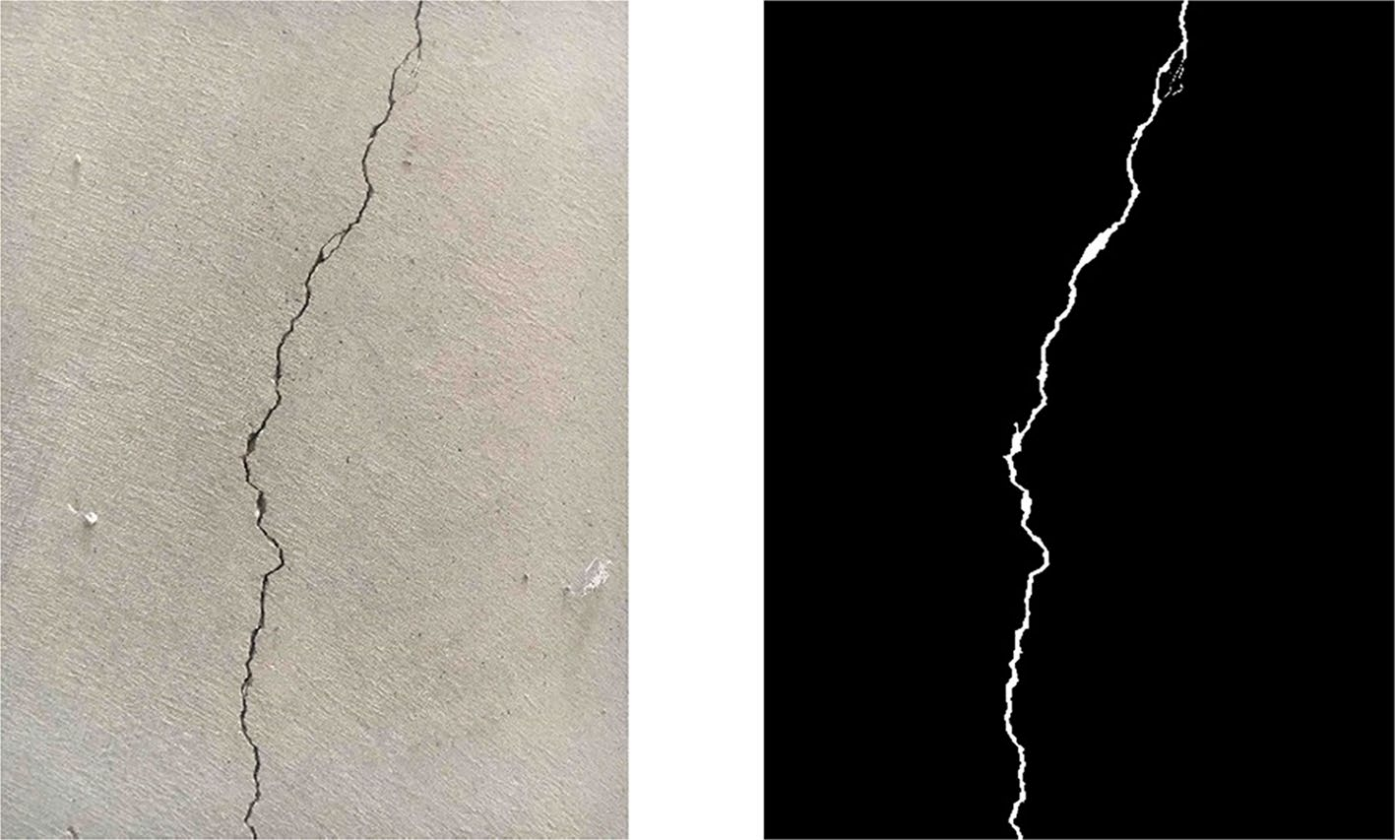
Sample picture of cracked concrete pavement along with its alpha map obtained from the dataset by Özgenel (2019).

Figure 1 demonstrates a sample picture obtained from the dataset along with its alpha map. The cracks in the concrete have a clear tendency to move upward in the direction from which they initialize, but a detailed breakdown of the movement pattern will be discussed in the following sections.

After the initiation of the crack, the latter may take any orientation based on the stresses existing in the concrete at the time of fracture. However, estimating those stresses is not easy and typically not possible without detailed knowledge of the construction and loading history of the concrete member. In most cases, cracks in concrete tend to propagate forward. This is because the tensile stresses in the concrete are highest at the tip of the crack. However, there are cases where cracks may propagate in other directions, such as sideways or backward. The path followed by a crack in concrete is not a straight line.

### Automatic crack detection and classification

#### Limitations of unsupervised crack detection

Given a database of images showing concrete surface cracks. Crack path tracking is the task of automatically detecting and following the path of cracks in concrete images. There are different ways to approach crack path tracking. One approach is to use a convolutional neural network (CNN) to detect and track the cracks in the images^18^. In this approach, a CNN is used to detect and track the cracks in the images. The CNN is trained first on a dataset of images containing cracks. Then, the CNN is used to detect and track the cracks in a new set of images. Other approaches include using an edge detector to find the cracks in the images and then tracking the cracks using a Kalman filter^19^.

Detecting cracks in pavement is important for safety reasons as well as for assessing the health of pavement. There are various techniques that can be used for crack path tracking^20,21^. The convex hull approach^22^ can be used to extract all the pixels in the image that belong to the crack and then draw the convex hull around the crack pixels. The algorithm is fast but fails to detect curved lines, and it is not very sensitive to small changes in the input image, which can lead to inaccuracies in line detection.

The marching cubes approach^23^ can be used to extract all the pixels in the image that belong to the crack and then construct a 3D mesh representation of the crack pixels. This approach has the advantage of being able to detect curved lines, and it is also less susceptible to noise and distortion in the input image. However, it is a more complex algorithm.

The Hough transform algorithm^24^ has been used in many applications, such as detecting straight lines in images and detecting circles in images. It is a robust and fast algorithm, which makes it suitable for real-time applications. Its application to tracking concrete cracks has been used in available studies such as^25^.

Automatic crack path tracing based on a database of images can have disadvantages^26,27^, such as:

- It may be difficult to accurately track the crack path, especially if it changes direction.
- The database may not contain images of all possible crack paths.
- The algorithm may not be able to accurately identify the crack path in all images.
- It may be difficult to generate a good database of images.
- The time required to track the crack path may be significant.
- The algorithm may be computationally expensive.

**Figure 2:**
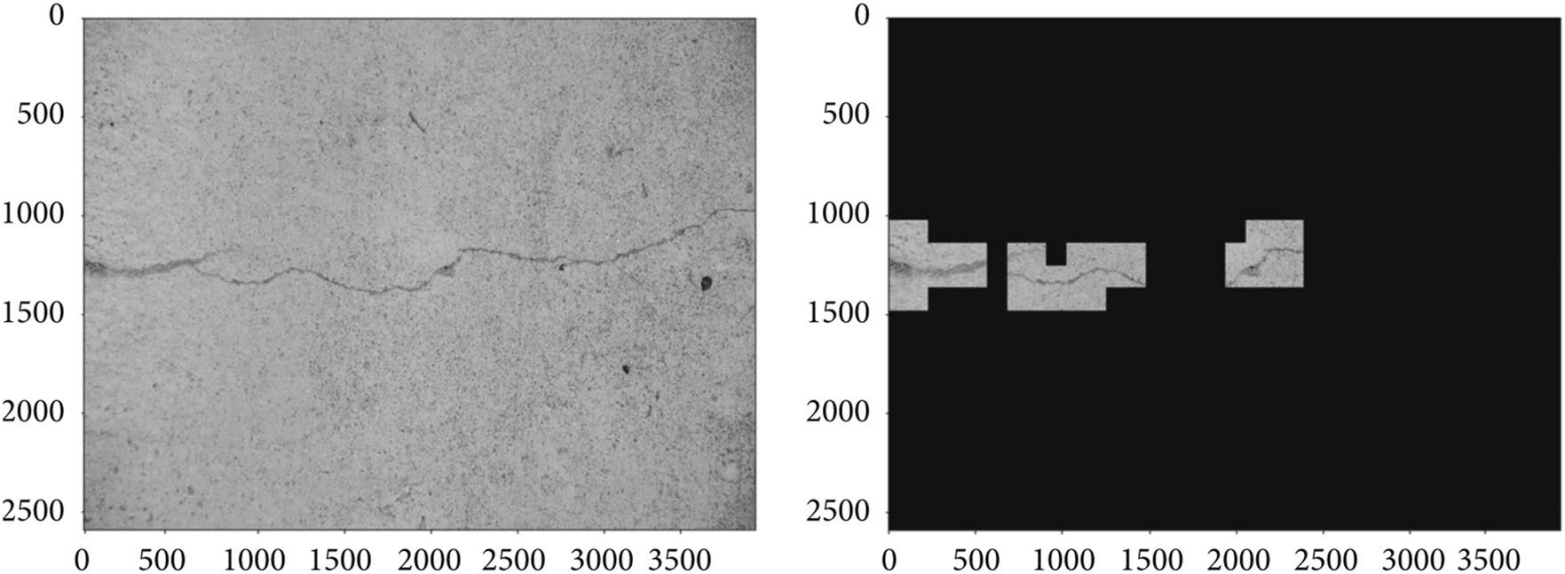
Failed automatic unsupervised crack detection (courtesy of^28^).

Figure 2 shows an example of a failed automatic tracing of a concrete crack path. The image was automatically traced using an improved algorithm based on a convolutional neural network^28^. The algorithm failed to detect some of the cracks in the image. This can lead to inaccurate results, which can be problematic for engineering applications.

**Figure 3:**
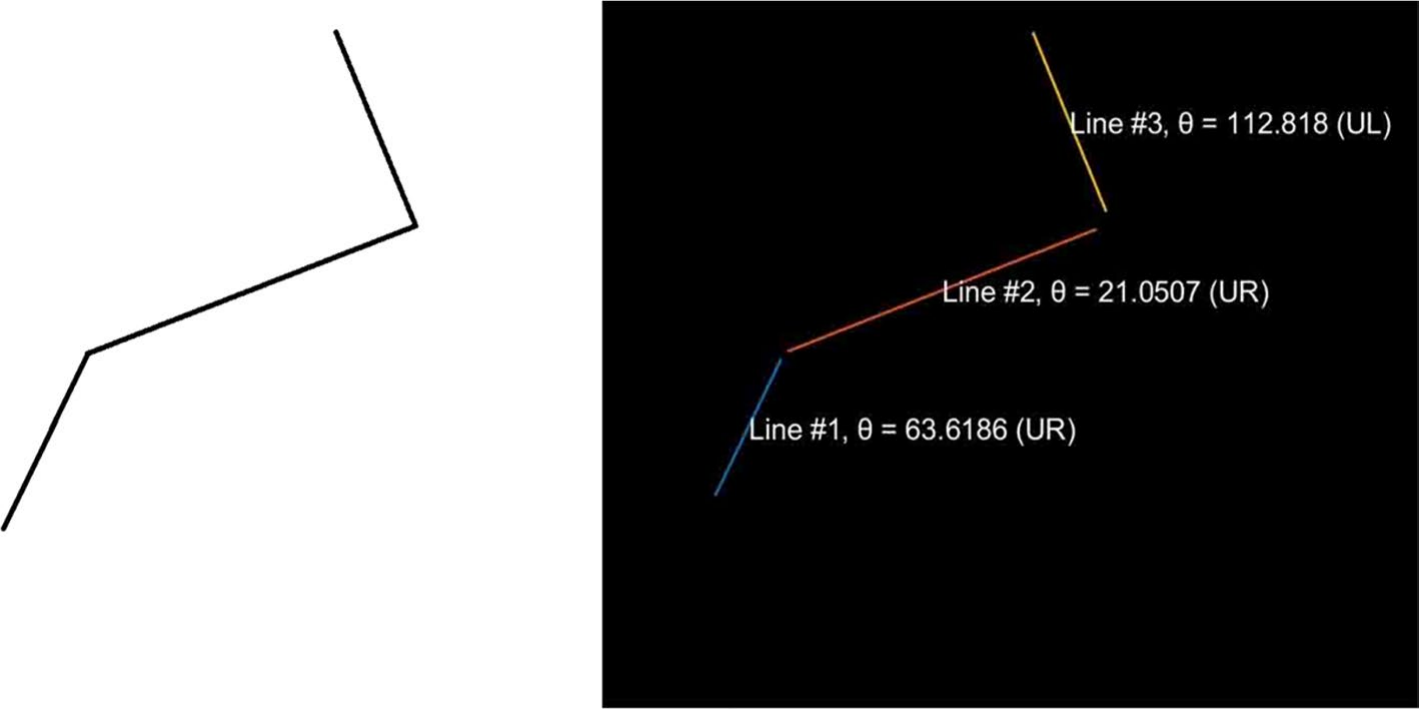
Example of successful Hough transform line detection.

**Figure 4:**
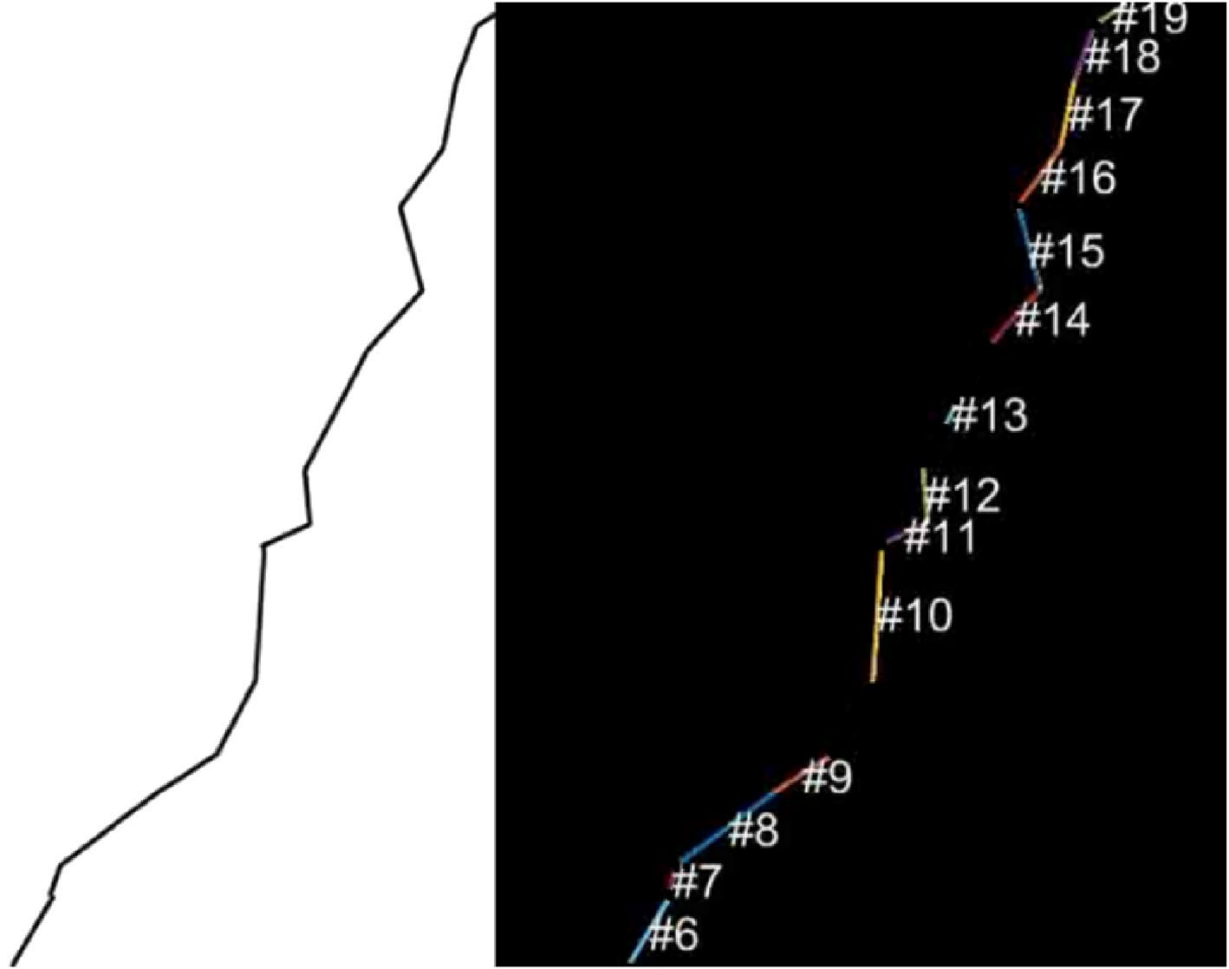
Example of failed Hough transform line detection.

Due to the nature of the present study, the required number of traced cracks is high; hence, an attempt was made to apply automatic crack detection and tracing. The first step in detecting the crack direction changes and their frequencies is to acquire a suitable dataset. Once the data were obtained, we used MATLAB^29^ with its built-in functions. The next step is to use a Hough Transform. For a simple artificial crack, such as the one shown in Fig. 3, the detection can be considered adequate. However, for more complex cracks where the crack path keeps changing direction very frequently with varying lengths, the detection algorithm starts missing some lines (see, for example, the artificial crack and its detection in Fig. 4).

Hough transform parameters such as the threshold, the minimum line length, and hood size were intensively modified to improve the accuracy of the detection. However, the results indicated that a set of parameters that suit a certain crack image may not suit another image, and more certainly, they would not suit all 100 pictures. As a result, it was concluded that the use of the Hough transform would not be sufficiently accurate for this study.

#### Interactive supervised crack pattern tracing

Given a database of concrete crack images, manual tracing of concrete cracks can have several advantages over automatic tracing, such as the following:

- Can be used to correct or improve the results of an automatic tracing algorithm.
- Can be used to identify and trace smaller cracks that may be missed by an automatic tracing algorithm.
- Can be used to trace cracks that are not parallel to the image plane, which may be difficult or impossible to do automatically.
- Can be used to correct or adjust the orientation of cracks in the image.

Despite the time-consuming and labor-intensive work involved, manual tracing is often still the best option for accurately tracing cracks in concrete images, especially in our current investigation, which requires very accurate crack line detection and correct path orientation.

**Figure 5:**
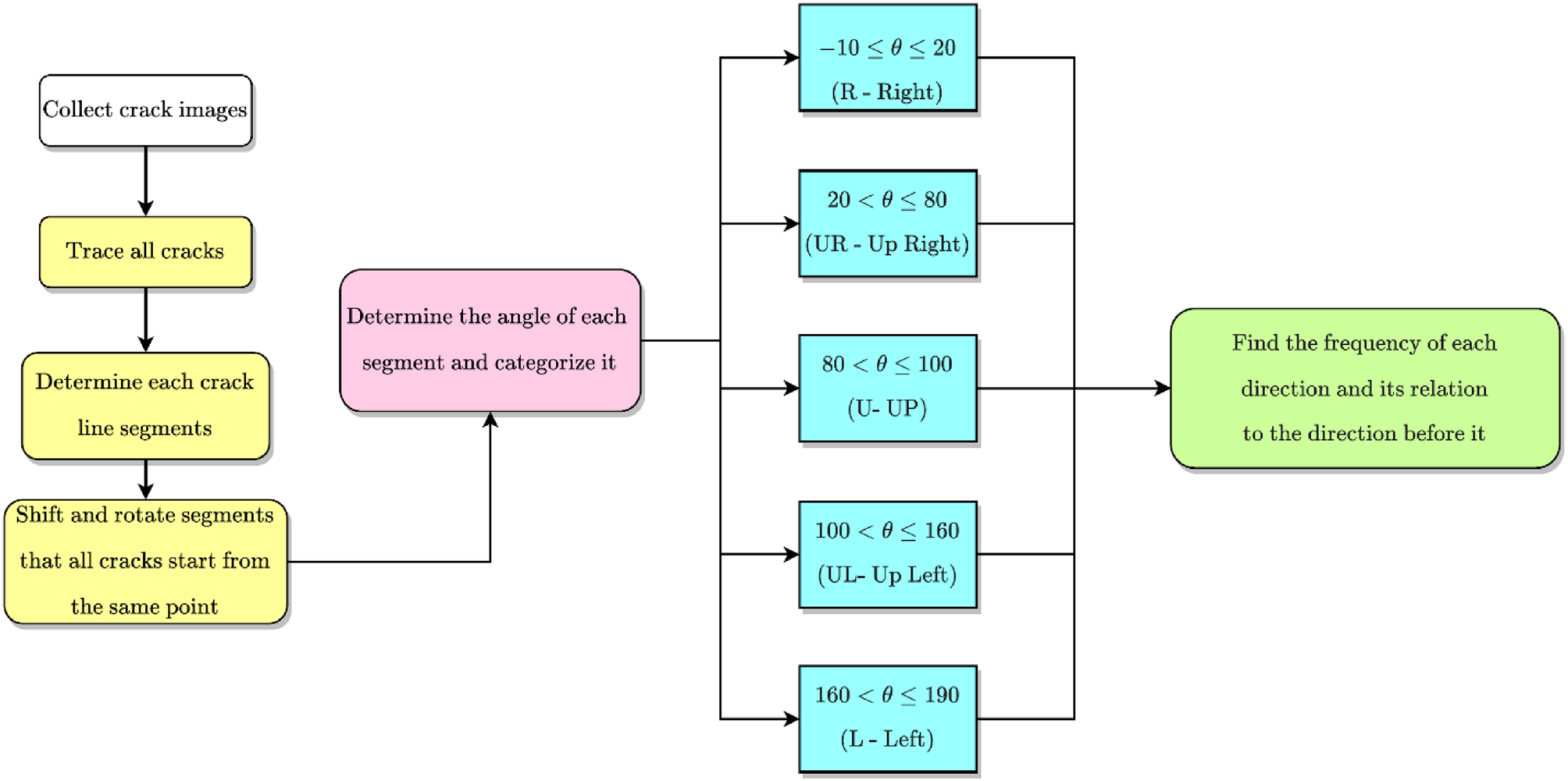
Flowchart of the developed crack tracing and analysis algorithm.

**Figure 6:**
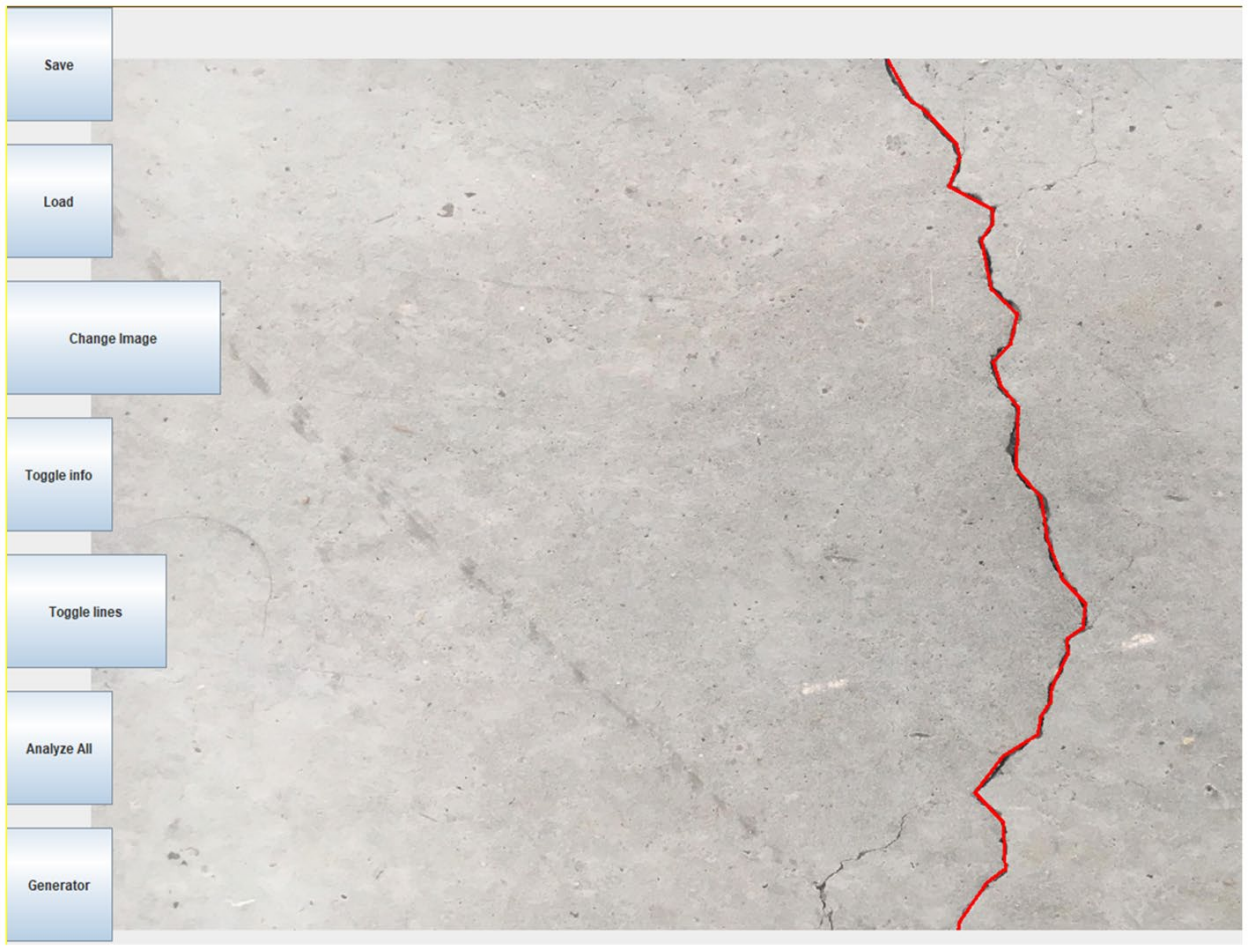
Tracing/analysis software developed.

The flowchart in Fig. 5 shows the algorithm developed for tracing and classification of concrete crack segments manually based on the database of images. A Java computer software with a user interface (Fig. 6) was developed to for an interactive crack tracing and classification. The program is designed to allow for higher levels of accuracy in crack pattern detection and works by internally tracing each line segment of the crack and collecting data of path coordinates and direction. This information is then post-processed by the same software to determine the crack pattern and the probability of the crack moving in a certain direction. The software stores a database that contains the $\left( {x,y} \right)$ coordinates of the points connecting each segment of the concrete crack, as well as the length of the crack, in addition to other relevant data related to the probabilities.

**Figure 7:**
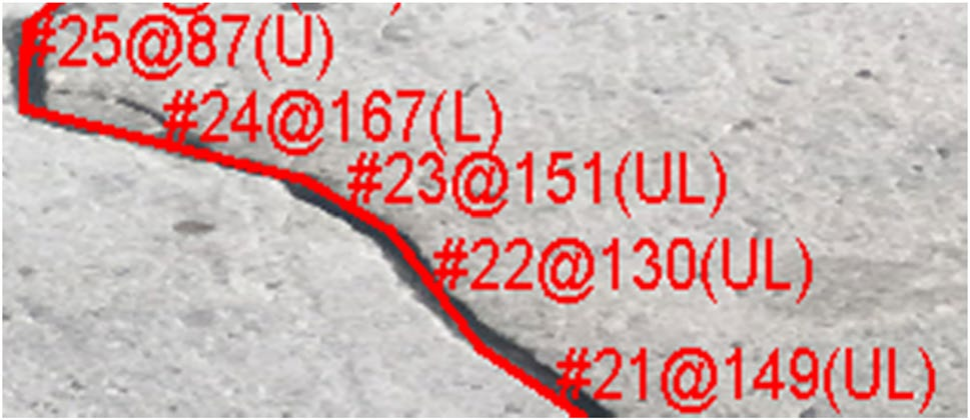
Example of a traced crack with the relevant information to be saved in a database.

The image shown in Fig. 7 is an example of a crack in a concrete surface that has been traced using the developed software.

## Probabilistic analysis of crack propagation in concrete surfaces

Mathematical models can be used to simulate the propagation of a crack in concrete^30,31^. As stated earlier, crack propagation in concrete surfaces can be considered a Markov chain, where the states are the different locations on the surface and the transitions are the propagation of a crack from one location to another. The problem that remains is to find the most likely path of the crack. There are a few different ways to approach this problem. One way is to use probabilistic approaches based on a large database of images of cracks in concrete surfaces. Then, using these probabilities, one can calculate the most likely path of the crack. Once the most likely path of the crack has been found, the next step is to determine the probability that the crack will reach a certain location. This can be done using computer simulations based on the computed probabilities of a crack segment takin on a certain direction.

The most likely path of the crack can be used to predict:

- The damage caused by the crack.
- The time it will take for the crack to reach a certain location.
- The size of the crack at a certain location.
- The shape of the crack at a certain location, etc.

To determine the probability that a crack will reach a certain location, we start with a set of initial conditions for the crack. We then calculate the most likely path of the crack using, for example, a Markov chain. We then calculate the probability that the crack will reach the desired location. There are a number of different models that can be used for crack propagation, and the most appropriate model will depend on the specific situation. Some common models include the random walk, the Wiener process, and Brownian motion.

The random walk model is the simplest and assumes that the crack progresses at a random, unpredictable rate.

Previous work on the random nature and probability of propagating concrete cracks is scarce, especially probabilistic methods. Some possible methods include studying the propagation and orientation of concrete cracks under controlled laboratory conditions, using mathematical models to simulate the propagation and orientation of concrete cracks, and studying the behavior of concrete cracks in real-world scenarios. However, due to the nature of concrete as a heterogeneous material and its related uncertainties, the best way to study the random nature and probability of propagation and orientation of concrete cracks is through computer simulations implementing some sort of stochastic process and with probabilities determined based on real-life database of crack images.

### Computation of crack orientation probabilities

As the propagation of cracks in concrete can depend on a variety of factors, such as the composition of the concrete, the loading conditions, and the environment, making deterministic predictions on cracks and their orientation is not possible. The propagation of cracks in concrete can be a very random process, depending on the factors mentioned above. For example, the composition of the concrete can affect the rate at which cracks propagate, as different types of concrete can have different levels of strength and flexibility. Additionally, the loading conditions on the concrete can also affect the rate of crack propagation, as higher loads can put more stress on the concrete and cause cracks to spread more quickly. Finally, the environment in which the concrete is located can also play a role, as factors such as temperature and humidity can affect the rate of crack propagation. Therefore, it is safe to say that the propagation of cracks in concrete can be considered a random walk, as it is affected by several uncertain factors.

A random walk has been used to model the probability of propagation and orientation of cracks in concrete^3,11^. The approach can be summarized in the following step-by-step algorithm:

*Input*: Image database of concrete surface cracks.

*Output*: $\left( {x_{i} ,y_{i} } \right)$ coordinates of the concrete cracks and the probabilities of the crack moving in any direction.

1. Load the image database
2. For each image in the database:
  - Extract the coordinates of the cracks in the image
  - Calculate the direction (i.e., angle) of the crack from one point to another
  - Calculate the frequencies of moving from one direction to another
3. Develop a Markov chain based on the probabilities of a crack moving in each direction

**Figure 8:**
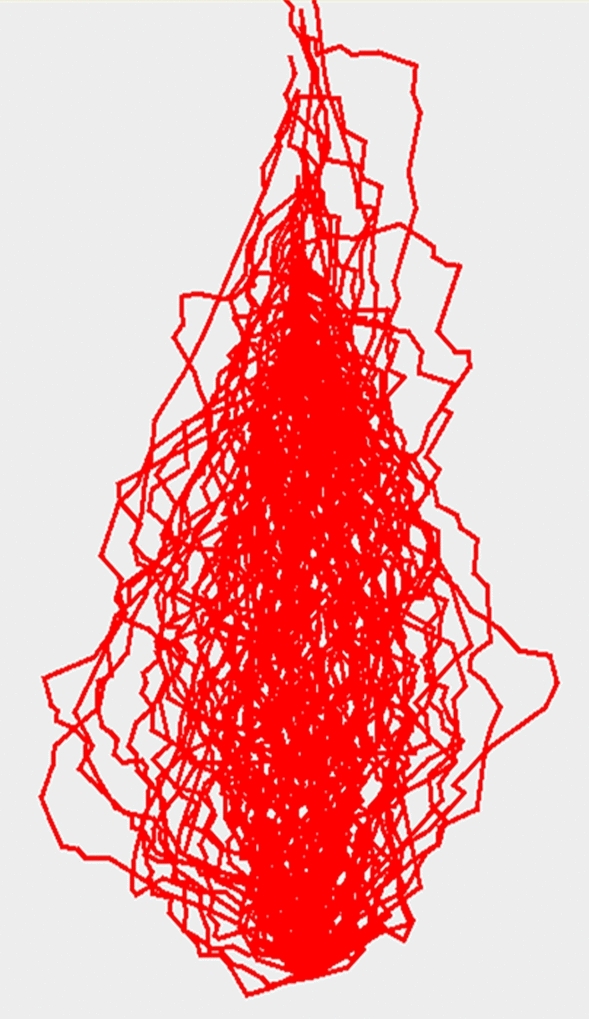
Cracks traced using 100 real images of cracks in different concrete pavement surfaces.

The developed program was used in tracing 100 cracked concrete pavements from the image database, and the results are shown in Fig. 8.

The resulting $\left( {x,y} \right)$ coordinates of each crack edge were used to detect the direction of each crack segment from one point to another. The crack is considered to be a polyline made up of several segments of different sizes. The output file contains the coordinates of the first point of the crack: $\left( {x_{1} = 0, y_{1} = 0} \right)$ then the $\left( {x_{i} , y_{i} } \right)$ coordinates of the subsequent points of the crack polyline. In addition, the direction of the crack from $\left( {x_{i} , y_{i} } \right)$ to $\left( {x_{i + 1} , y_{i + 1} } \right)$ was post-calculated.

In the present study, the five possible directions/orientations of a crack segment are north (or forward/up); west or right); east (or left); northwest (up-right); and northeast (up-left).

The classification of the crack segment direction is based on a simple approach involving the calculation of angles. The flowchart of Fig. 5 shows the details of this process.

### Random walker #1

Random Walker # 1 (model 1) is the simplest of the two models developed in this study. The possible directions of a crack segment are left, up, or right. As stated before, all concrete cracks have the tendency to move forward; therefore, the “downward” direction of a crack is not acceptable. Based on the frequencies of moving from one direction to another, we calculated the probabilities. A Markov chain was then developed.

**Figure 9:**
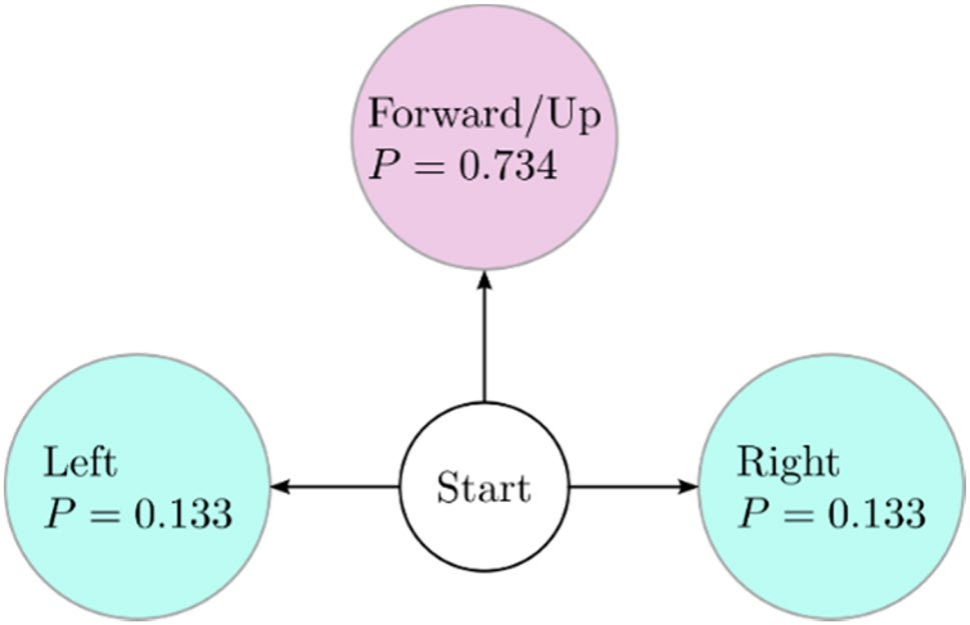
Random walker #1: crack orientations and their corresponding probabilities.

In model 1, the probability of a crack taking a direction is assumed to be independent from the past crack direction. Thus, at any point in time, moving in a certain direction will have the same probability. Based on this assumption, the probabilities of the crack moving right, up, and left are 0.133, 0.734, and 0.133, respectively. Figure 9 illustrates this information visually. As we can see, the crack moving in the upward direction has the highest probability, and the crack moving to the left and right share similar but smaller probabilities.

**Figure 10:**
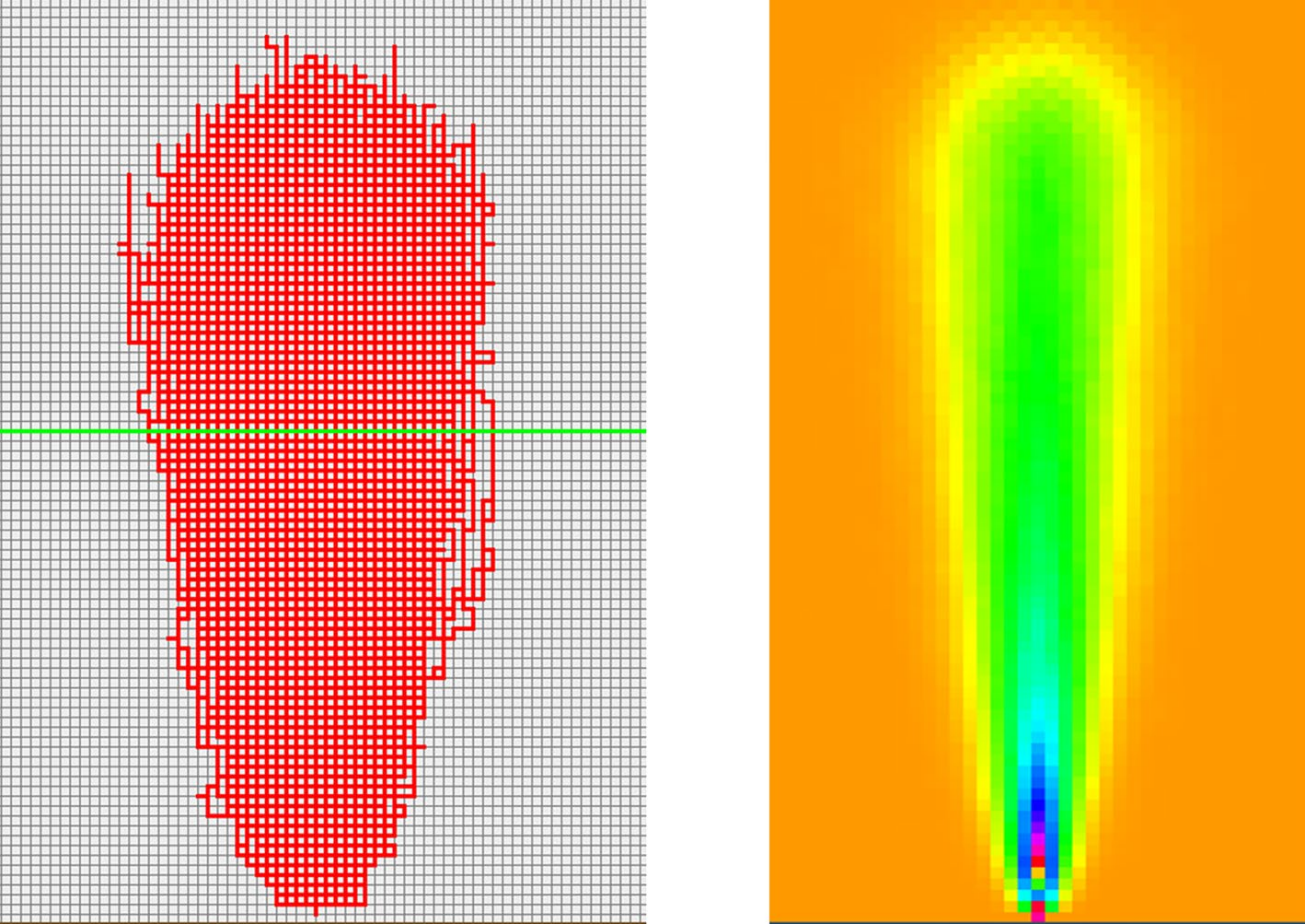
Random walker #1 simulated cracks.

Next, using the Markov chain in Fig. 9 and starting from the point with coordinates (0,0), thousands of cracks were simulated. The results are shown in Fig. 10. The red lines are the paths that the cracks are most likely to follow based on the computed probabilities.

Again, the results of the simulation show that the crack is most likely to propagate in a vertical direction. However, it is important to note that the results are not 100% accurate and should be used as a guide only.

At every $y$-level, the probability of having a crack passing by that level is:

1 $$ P\left( {x,y} \right) = \sum p\left( {x,y} \right) \times p\left( x \right) \times p\left( y \right) $$

where $p\left( {x,y} \right)$ is the probability of a crack passing by point $\left( {x,y} \right)$, $p\left( x \right)$ is the probability of a crack passing by point $\left( {x,y} \right)$ in the vertical direction, and $p\left( y \right)$ is the probability of a crack passing by point $\left( {x,y} \right)$ in the horizontal direction.

The distribution of cracks at any level of coordinate $y$ is found to be normally distributed, as shown in Fig. 10. Repeating this process for different $y$-levels, we obtain a series of histograms resembling the normal distribution. However, these probability distributions have almost the same mean centered around $x = 0$ but increasing standard deviations as we move up the $y$ ordinate axis away from where the crack has initiated (i.e., $\left( {x = 0,y = 0} \right)$). The means represent the $x$-coordinates of the crack, while the standard deviation represents how far the crack has moved away from the mean, either to the left when negative or to the right when positive. The mean value is the average $x$ positions of cracks at a specific $y$-level, while the standard deviation is a measure of how dispersed the cracks are around the mean. This information can be used to determine the shape and behavior of the cracks.

The heatmap shown in Fig. 10 provides a visual representation of the data; it can be used to better understand the behavior of the cracks. Observe that the cracks move mostly upward, with a very small deviation to the right of the centerline accompanied by a slight reduction in the likelihoods. The shape of the cracks is heavily centered because of the equal right and left crack orientation probabilities.

**Figure 11:**
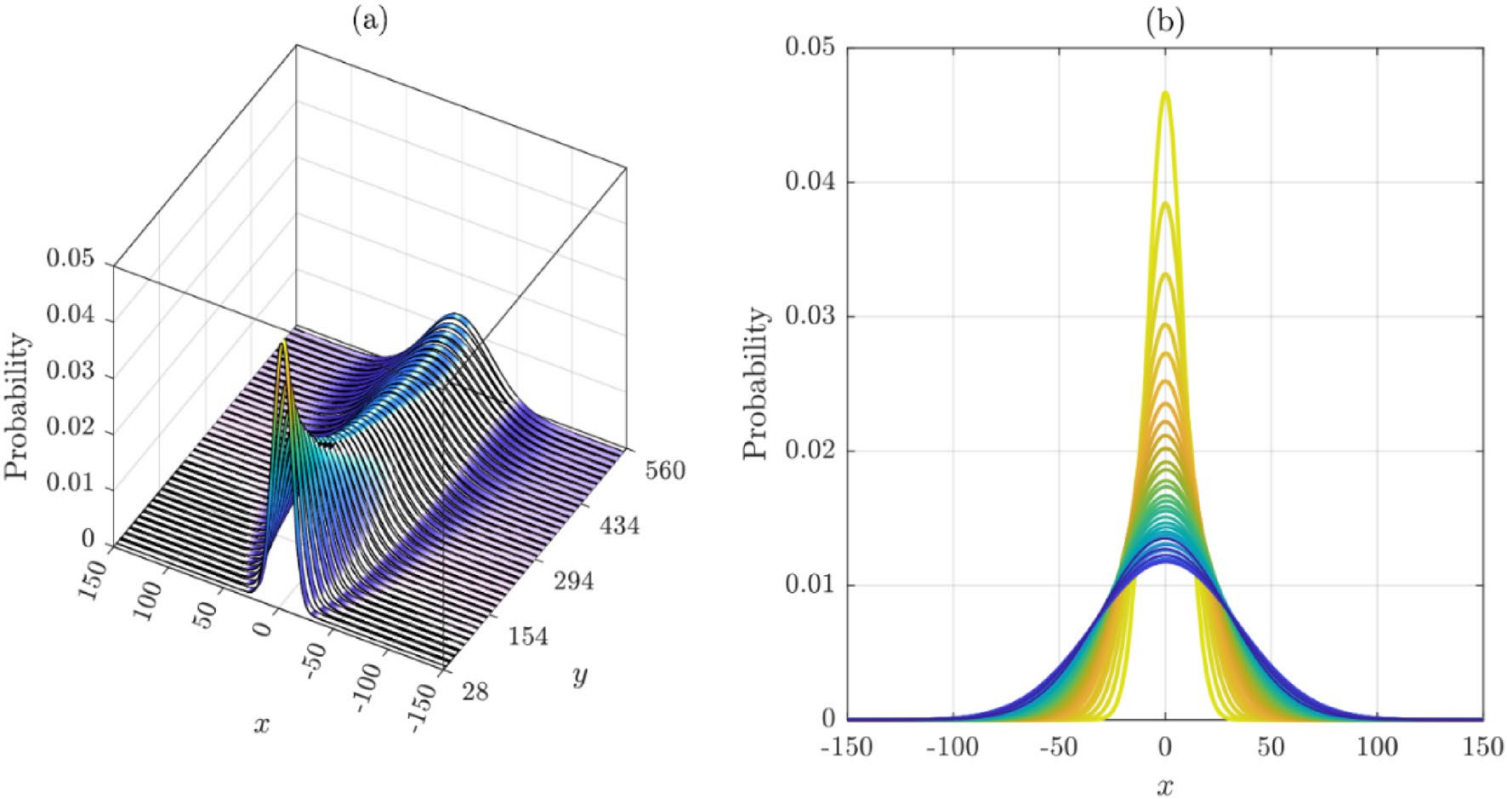
Probability distributions of crack distribution at different $y$-levels (Random Walker #1): (**a**) Ribbon-plot view; (**b**) 2D congregated curves.

Figure 11 shows histograms of crack distributions at different $y$-levels selected randomly from different heights of the concrete surface. The histograms clearly depict typical Gaussian distributions with very close means, thus confirming the previous predictions. The standard deviation, however, differs from one level to the next. Moving up the $y$-axis of the concrete surface, the standard deviation increases, depicting a clear diffusion process.

### Random walker #2

**Figure 12:**
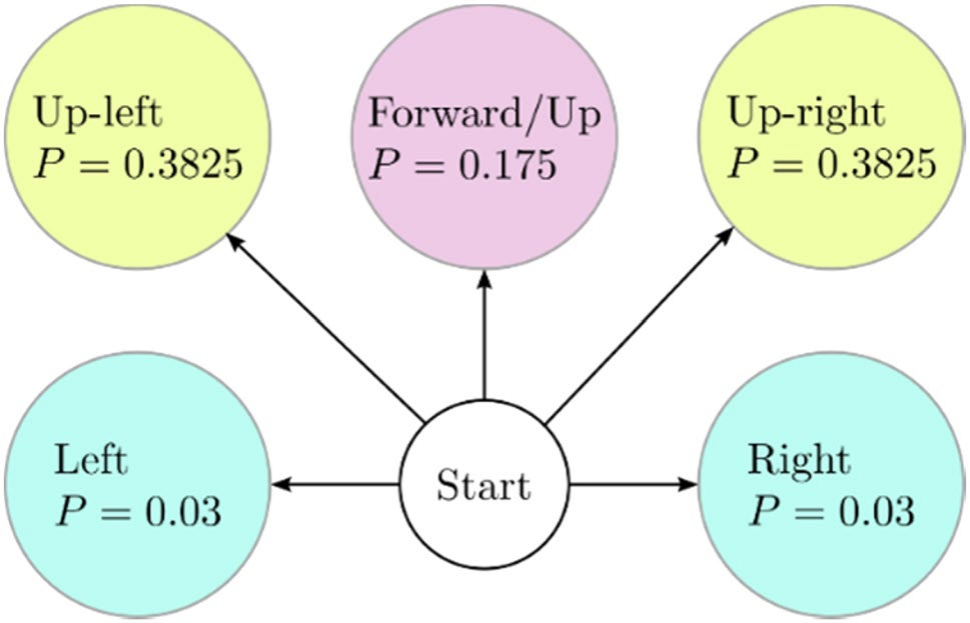
Random walker #2: crack orientations and their corresponding probabilities.

In model 2, more possible crack orientations are considered: right, up-right, forward/up, up-left, and left. Analyzing the cracks from the image dataset, we determined the probabilities of the crack moving to the right, up-right, up, up-left, and left as 0.030, 0.3825, 0.175, 0.3825, and 0.030, respectively. Figure 12 illustrates this information visually as a Markov chain.

**Figure 13:**
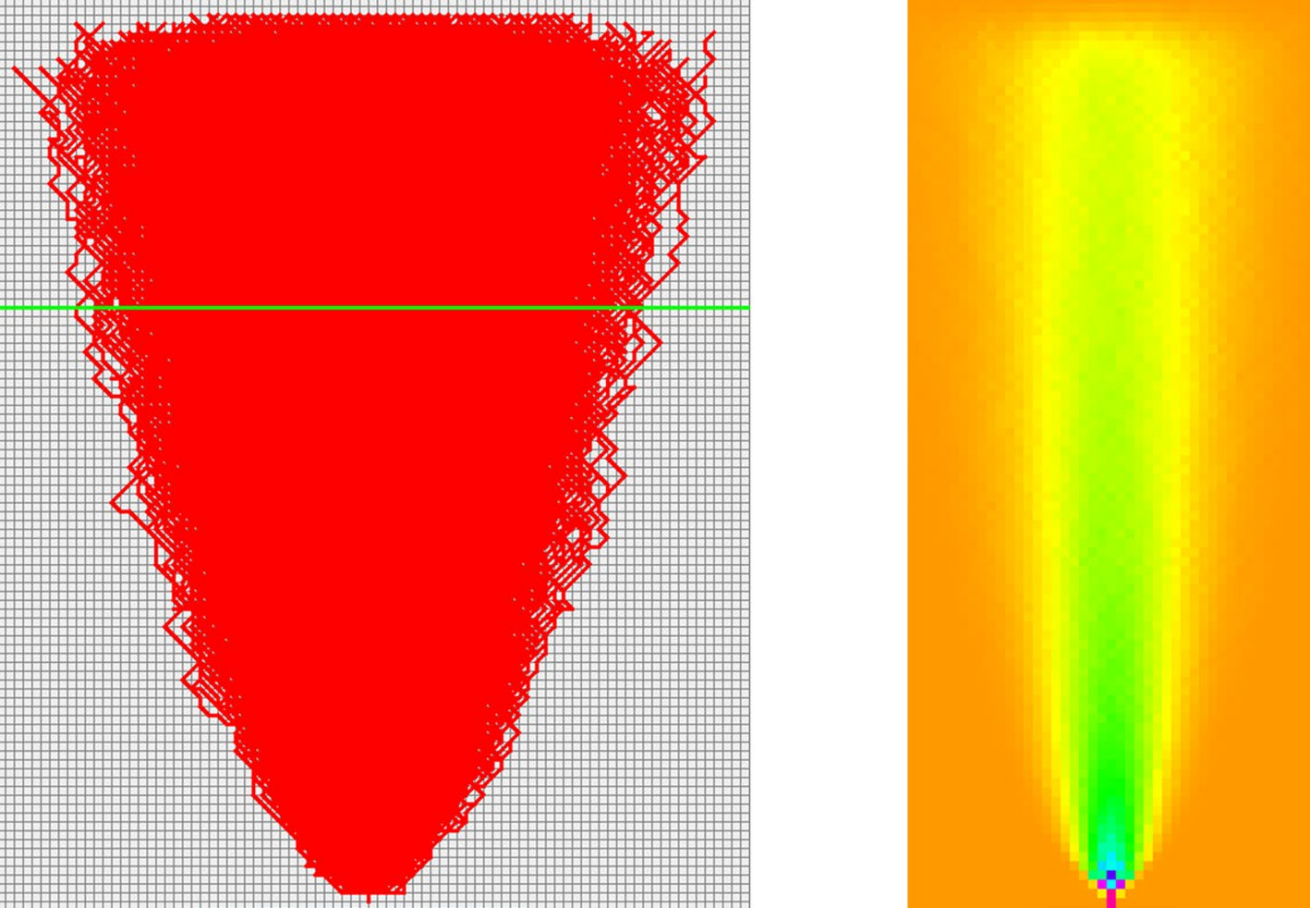
Random Walker #2 simulated cracks.

A total of 100,000 cracks were simulated based on the computed probabilities (see Fig. 13). It is clear that the distribution of the cracks maintained a normal distribution at every $y$-level. Similar to model 1, the mean of the distributions is mostly centered around $x = 0$, however, accompanied by noticeable reduction in the likelihoods, especially that of the mean. The standard deviation starts from essentially $x = 0$ at the point of crack initiation and increases from there all the way up the $y$ axis, indicating that the cracks are spreading more, thus depicting a clear diffusion process. There was, however, a small deviation of cracks from the vertical centerline ($x = 0$) toward the right.

**Figure 14:**
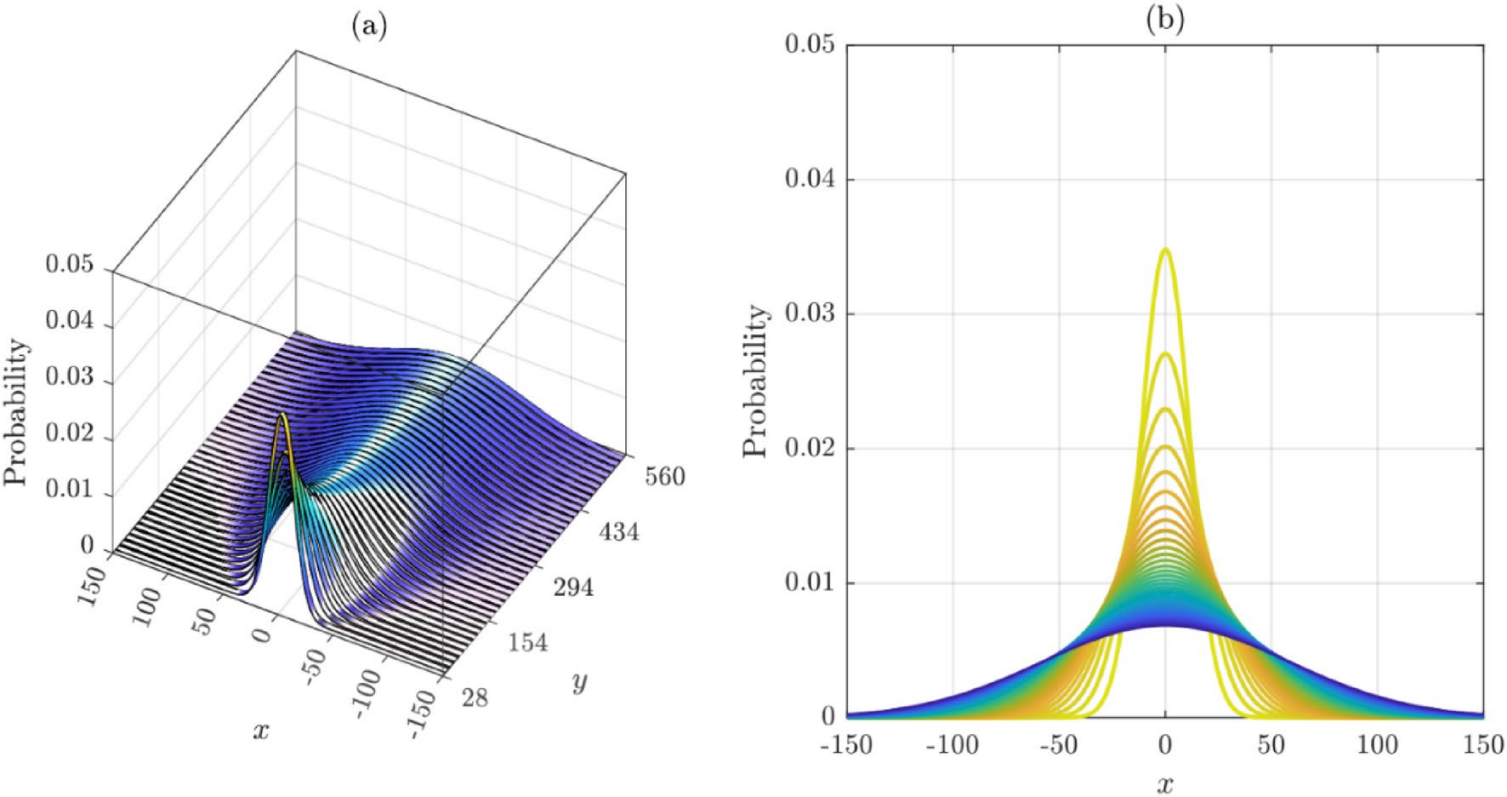
Probability distributions of crack distribution at different $y$-levels (Random Walker #2): (**a**) Ribbon-plot view; (**b**) 2D congregated curves.

The histograms of crack distribution at different levels in the $y$-axis are normally distributed, as shown in Fig. 14. Repeating this process for different y-levels, we obtain a series of similar-shaped histograms. The statistical properties of the normal probability distributions corresponding to each of these histograms share approximately similar means but different standard deviations. It should be emphasized that the mean is represented by the $x$-coordinate of the crack edge, while the standard deviation represents how far the crack has moved away from the mean (i.e., left or right of the centerline at $x = 0$).

Similar to model 1, in model 2, the direction a crack takes in the next step/time is determined by the current step only, suggesting that the process has the Markovian property.

Model 2 represented by the current Random Walker #2 may be considered more representative of the real behavior of the crack propagation of cracks on concrete pavements and surfaces. Of course, more complicated random walk models can be engineered to go deeper into the behavior of each crack segment orientation following a series of previous states. However, these models will likely lose the Markovian property and hence represent a totally new stochastic process.

### Analysis of the behavior of the random walkers

**Figure 15:**
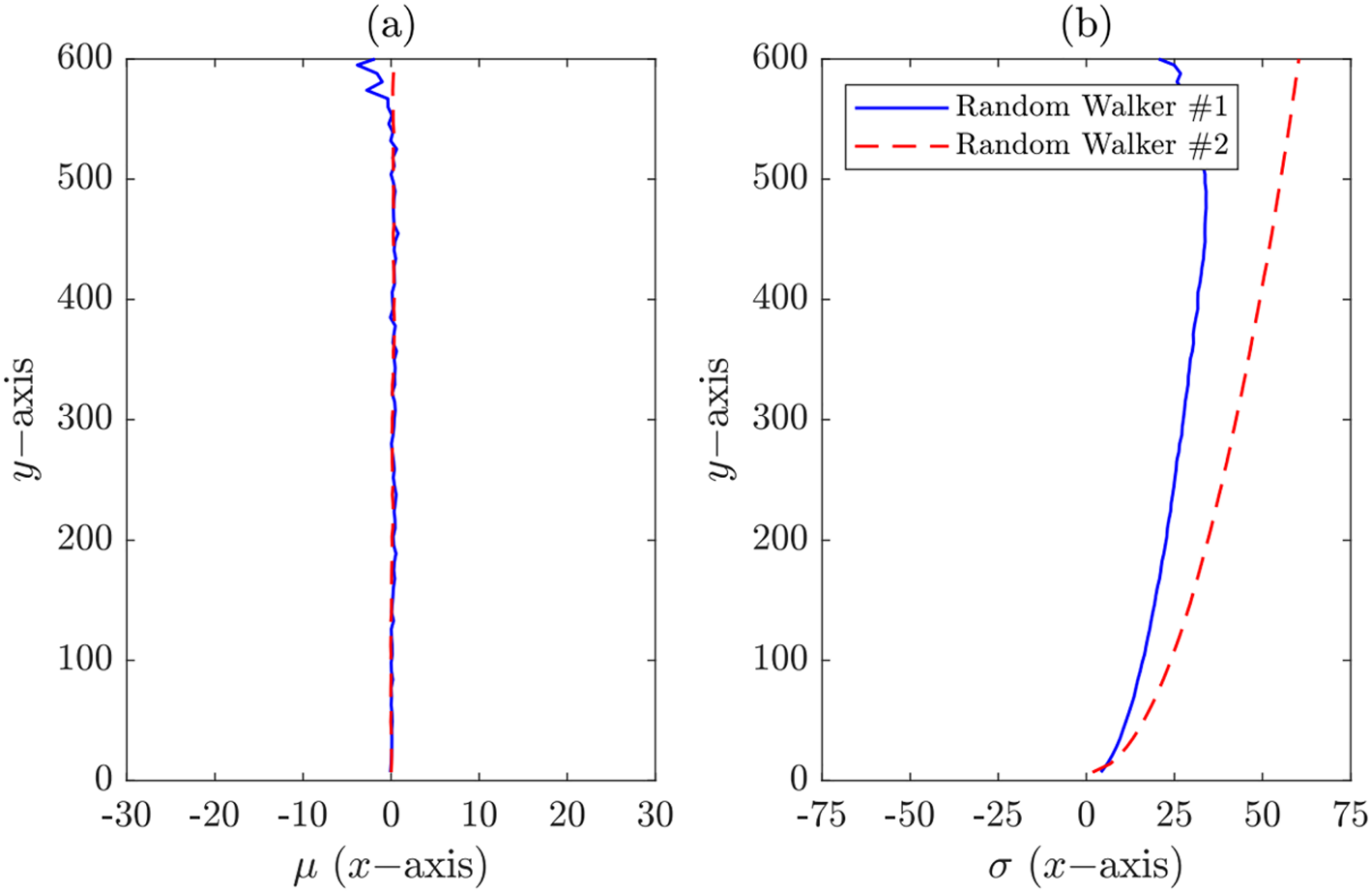
(**a**) Mean and (**b**) standard deviation (only $\sigma^{ + }$ shown) of probability distributions at different $y$-levels.

As demonstrated before, models represented by random walkers #1 and #2 are unbiased (see Fig. 15a).

Even though there was no drift of means of crack positions represented by the unbiased random walker #2, the standard deviation did actually drift to the right of the centerline ($x = 0$) (Fig. 15b). The standard deviation increases as we move up the $y$-axis from the origin, suggesting that the concrete surface cracks have the tendency to spread as the crack propagates. More precisely, the likelihood of finding cracks at positions away from the $x = \pm \sigma$ is lower than at positions within the interval $\left[ { - \sigma , + \sigma } \right]$.

## Discrete probability distribution of concrete surface cracks

In this section, we are interested in investigating the probability distribution of cracks passing by a position $\left( {x,y} \right)$ at any time, $t$. First, we present the mathematical foundations of the discrete probability distribution of concrete surface cracks. Then, we define failure (i.e., worst case) of concrete surfaces due to the propagation of cracks. Finally, a theorem is proposed with its proof.

### Master equation

Starting with a random walker similar to #1 shown in Fig. 9. We will consider that the crack propagates in small jumps, ${\Delta }x$, in small time intervals, ${\Delta }t$, to the left and right with probabilities $p_{L}$ and $p_{R}$, respectively. In addition, the crack can also propagate upward with a small jump, ${\Delta y}$, in small time intervals, ${\Delta }t$, with probability $p_{U}$, such that $p_{L} + p_{U} + p_{R} = 1$.

The evolution of the probability distribution of cracks can be described by the following Master equation:

2 $$ P\left( {x,y,t + {\Delta }t} \right) = p_{L} \times P\left( {x - {\Delta }x,y,t} \right) + p_{U} \times P\left( {x,y + {\Delta }y,t} \right) + p_{R} \times P\left( {x + {\Delta }x,y,t} \right) $$

$P\left( {x,y,t} \right)$ is the probability of a crack passing by position $\left( {x,y} \right)$ at time $t$. At the next time step, ${\Delta }t$, the probability can be described by the Master equation with the initial condition:

3 $$ p\left( {x,y,0} \right) = \delta \left( {x,y} \right) $$

in which $\delta \left( {x,y} \right)$ is the Dirac delta function.

**Figure 16:**
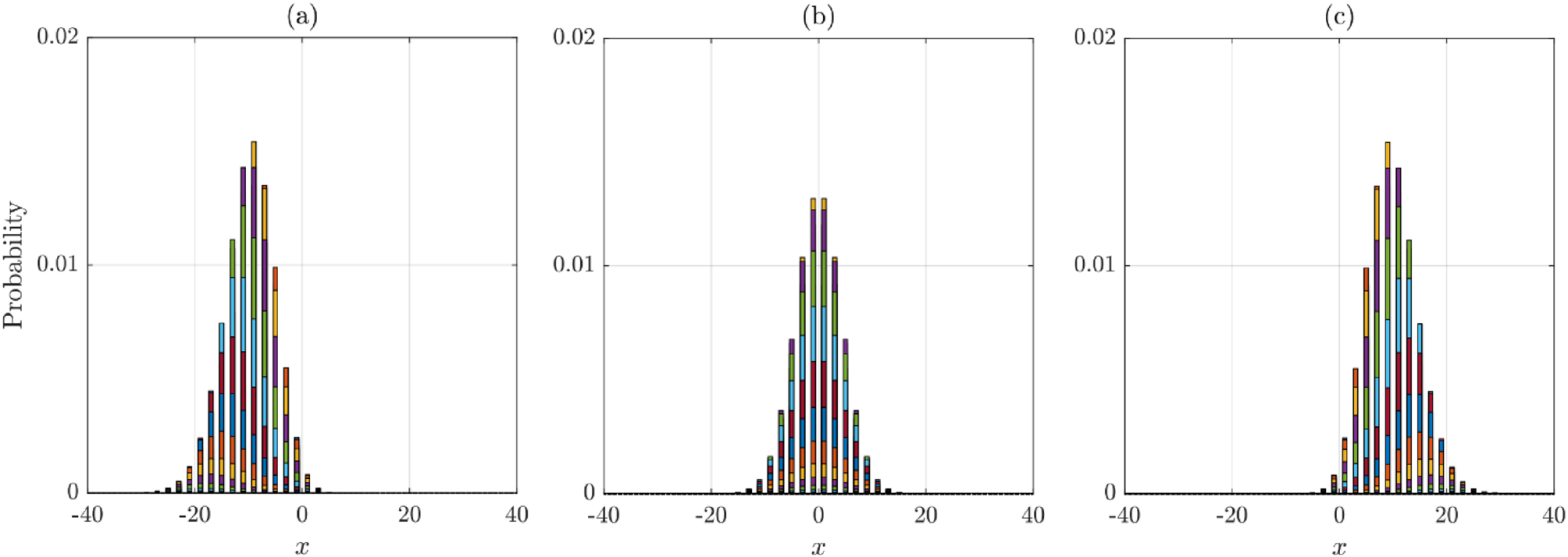
Solutions to the master equation of diffusion: (**a**) with left drift; (**b**) without drift; (**c**) with right drift.

Assuming generic values to the probabilities, time, and space steps, the numerical solution to the master equation is shown in Fig. 16. Clearly, the probability distributions of cracks are normally distributed and diffuse. There are three cases represented in the figure: probability of cracks drifting to the left due to $p_{L} > p_{R}$ (Fig. 16a), probability of cracks remaining in the center due to the equal probabilities of moving to the left and right (i.e., $p_{L} = p_{R}$ (Fig. 16a), and probability of cracks drifting to the right due to $p_{R} > p_{L}$ (Fig. 16c).

Without the loss of generality and relying on our engineering judgment, we will assume that the random walker is unbiased to move to the left or to the right such that $p_{L} = p_{R} = q$ and $p_{U} = 1 - 2q$; then, the Master equation reads:

4 $$ P\left( {x,y,t + {\Delta }t} \right) = qP\left( {x - {\Delta }x,y,t} \right) + \left( {1 - 2q} \right)P\left( {x,y + {\Delta }y,t} \right) + qP\left( {x + {\Delta }x,y,t} \right) $$

The probability distribution at any position and after the number of steps or time steps can be obtained by numerically solving equation Eq. (4).

### Probability of failure

We must first define what we mean by the “failure” of a concrete pavement surface, and then we present a theorem to estimate the probability of its occurrence.

#### Definition 1

**Figure 17:**
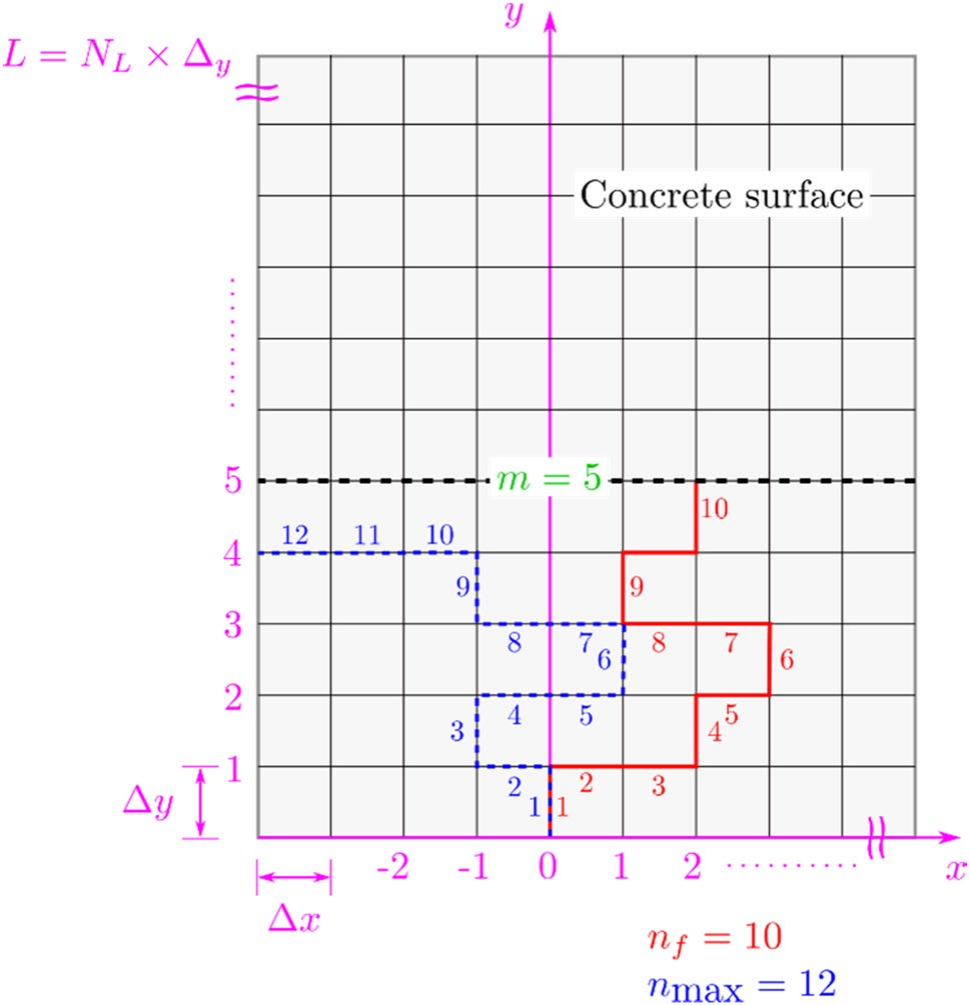
Definitions of $m$, $n_{f}$, $n_{{{\text{max}}}}$, and $N_{L}$.

(*crack segment*) A concrete surface crack segment is defined as a unit of crack (one walk for the random walker in any of its degrees of freedom) with length ${\Delta }x$ or ${\Delta }y$ (see Fig. 17), depending on the direction. When the concrete surface is divided into a grid, ${\Delta }x$ and ${\Delta }y$ are then space steps in the $x$, respectively, $y$, directions.

#### Definition 2

(*failure criterion*) Usually, failure would refer to the inability of the material to carry load or more generally, do its intended purpose. In the case of a pavement, this would refer to the inability of the pavement to provide a safe and comfortable platform for vehicles and people. Such state, of course, is not to be achieved, and thus maintenance is required beforehand. The question remains though as when should maintenance be conducted? We cannot afford to be doing maintenance every time a small hair-sized crack appears. And the answer to such question is a complicated one that depends on several factors such as the importance of the structure, the environment, and growth rate of the crack. Thus, it can be generalized that a concrete surface reaches the state of failure when the crack propagates at least $m$ forward crack segments along the considered length $L$ of the surface. When $L$ is divided into $N_{L}$ segments, then $m < N_{L}$. Both $m$ and $N_{L}$ are integer numbers (see Fig. 17).

#### Definition 3

($n_{f}$): The minimal number of crack segments leading to the failure is denoted by the integer $n_{f} \ge m$ (see Fig. 17).

#### *Example 1*

Consider a concrete pavement of length $L = 100$ divided into 100 segments. The surface may be considered to have reached the state of failure when $m = \frac{1}{3}L \approx 33\% L$ forward walks/crack segments. In this case, for practical reasons, an inspection of the status of the pavement is required to take place, at least, after $m{\Delta }t$ time. The time step ${\Delta }t$ can be 1 day, 1 week, or 1 month; the important is that it is a fixed time period selected by the inspection team according to the repair and rehabilitation standards of the state/municipality in control.

Note that the value of $m$ is dependent upon many factors, such as the material properties, concrete thickness, state of the concrete surface, weather conditions, and loading history; therefore, it is not easy to compute precisely. However, it can be assumed based on engineering judgment, design, expert opinion, or it can even be based on a probabilistic assumption.

#### Theorem

(probability of failure) *Given n crack segments: The concrete surface failure due to crack development and propagation has the probability *$$\left( {\begin{array}{*{20}c} n \\ m \\ \end{array} } \right)\left( {1 - 2q} \right)^{m} \left( {2q} \right)^{n - m}$$ *after* $n$ *crack segments *(*or walks*)* in any of the three degrees of freedom *(*left, right, and forward or up*).

#### *Proof*

According to the discrete probability model in Eq. (2), the model is considered trinomial; however, since, in the present case, the left and right probabilities are equal to the probability of crack propagation $q$, our model (Eq. (4)) can be considered as a binomial model with a success probability (i.e., reaching concrete failure in this case) of $\left( {1 - 2q} \right)$ and failure probability of $2q$. The success in this case is reaching the $m$ forward steps, which means failure in reality. $\square$.

#### Corollary

*According to the binomial distribution, the probability of success in reaching exactly* $m$ *forward steps, after* $n$ *trials* (*trials here means walks or crack segments in any of the three degrees of freedom*), *given the probability* $q$ *of moving left or right, is given by*:

$$ P\left( {X = m;n|q} \right) = \left( {\begin{array}{*{20}c} n \\ m \\ \end{array} } \right)\left( {1 - 2q} \right)^{m} \left( {2q} \right)^{n - m} . $$

Again, according to the binomial distribution, the expectation of the worst case, defined as a random variable $X$, occurring after $n$ crack segments, in any direction of the three degrees of freedom, is:

$$ E\left[ {X = m;n|q} \right] = n\left( {1 - 2q} \right), $$

and the variance is:

$$ V(X = m;n|q) = n\left( {2q} \right)\left( {1 - 2q} \right). $$

To reduce the probability of failure, the quantity $\left( {1 - 2q} \right)$ should be minimized. In other words, we have to find a way to force the cracks to take the left and right directions more than the forward direction. One of the many ways to achieve this is by incorporating fibers with a certain amount at targeted locations. Adding fibers to concrete makes Fiber Reinforced Concrete (FRC). Nevertheless, the main problem that remains unanswered is the estimation of the probability $q$. This probability is a function of many variables, such as the material properties, concrete surface state, and thickness. However, it can be estimated by engineers after a number of observations of crack development over a finite period of time.

#### *Example 2*

Consider again Example 1. The probability of a crack reaching exactly $m = 33$ forward walks after 37 random walks in the three degrees of freedom with probability $q = 0.05$ is computed using the binomial probability model as $$P\left( {X = 33;n = 37|q = 0.05} \right) = \left( {\begin{array}{*{20}c} {37} \\ {33} \\ \end{array} } \right)\left( {1 - 0.1} \right)^{33} \left( {0.1} \right)^{4} = 0.204$$. This means that the probability of reaching failure exactly after 37 ${\Delta }t$ time units is 20.4%.

The expectation can also be calculated as $E\left[ X \right] = 37\left( {1 - 0.1} \right) = 33.3$ with a variance of $V\left( X \right) = 37\left( {0.1} \right)\left( {1 - 0.1} \right) = 3.33$. This example suggests that there is a 20% chance of concrete failure due to crack propagation in 37 ${\Delta }t$ time (assuming a crack segment takes on average ${\Delta }t$ time unit to propagate).

The probability of exceeding $m$ can also be calculated using the binomial distribution, $P\left( {X \ge 33;37{|}0.05} \right) = 0.691 \approx 70\%$. This means that after 37 random walks, there is a 70% (or high chance) of concrete failure due to crack propagation.

#### *Example 3*

Assuming in this example that the pavement has been engineered well such that the cracks that develop can move either left or right with a probability $q = 0.4$ (or 0.2 probability of the crack propagating one segment forward), in this case, the number of random walks needed to reach failure with the same 70% probability is 176 walks. In other words, it would take the pavement much longer time (approximately 4.8 times) to reach the failure state.

The above examples demonstrate a possible way to inspect and rehabilitate concrete pavements based on probabilistic approaches. Making inferences on the probability quantity $q$, the team monitoring the health of the pavement needs to schedule their site visits and conceive the pavement rehabilitation and maintenance strategies. Note that a Bayesian approach can be used to update the value of $q$ after each site visit and then update the schedules and maintenance strategies as a result.

### Numerical simulations

In this section, we will run computer simulations to first numerically verify the probability of failure theorem and then to make some inferences. We consider several cases with different probabilities of cracks taking one of the three directions: left, right, and forward, with the probabilities $q$, $q$, and $1 - 2q$, respectively.

There are two limiting cases:

1. When $q = 0.0$, the crack will propagate solely in the forward direction. In this case, $n_{f} = m$. $n_{f}$ is defined in Definition 3.
2. When $q = 0.5$, the crack propagation is a symmetric random walk where only the left and right directions are allowed for the crack to move. In this case, $n_{f}$ is undefined (i.e., it takes forever for the concrete surface to fail.)

The other cases to be considered in the next numerical simulations include the vector $q = \left\{ {0.05, 0.1, 0.17, 0.25, 0.30, 0.35, 0.40, 0.45, 0.49} \right\}$. The scenarios covered by these cases are inclusive and serve to answer some important questions, such as what is the probability of failure? and how long does it take for failure to occur? The answers to such questions have implications in the evaluation of the concrete surface condition, inspection schedule, and rehabilitation/reconstruction techniques.

The developed software was further developed to include the capability to have as input a vector $q$ and $N$ simulations, $M$ number of times, and output the number of failures after each simulation and how long it takes to reach failure (i.e., $n_{f}$), if any. After the initiation of an artificial crack, the number of crack segments allowed for the crack to propagate before stopping is $n_{{{\text{max}}}}$.

In the present numerical simulations, $N = 1000$ full cracks of length equal to $n_{{{\text{max}}}}$ = 300 (see Fig. 17 for a pictorial definition of $n_{{{\text{max}}}}$) and each full crack is re-simulated $M = 500$ times. Therefore, in total, there are $M \times N = 500 \times 10^{3}$ artificial cracks.

As per Definition 2, we chose $m = \frac{{N_{L} }}{3} \approx 33$ forward cracks to reach the state of failure. The results of numerical simulations are reported in Table 1.

**Table 1:** Results of numerical simulations.

$q$	#Fails/5E5	$\mu_{{n_{f} }}$	$\mu_{{n_{s} }} = \mu_{{n_{f} }} - m$	$P_{f} = 1 - \frac{{\mu_{{n_{s} }} }}{{\mu_{{n_{f} }} }}$	$1 - 2q$
0.0*	1	33	0	1.000	1.000
0.05	1	37	4	0.892	0.900
0.10	1	41	8	0.805	0.800
0.17	1	50	17	0.660	0.660
0.25	1	65	32	0.508	0.500
0.3	1	81	48	0.407	0.400
0.35	1	108	75	0.306	0.300
0.4	1	161	128	0.205	0.200
0.45	0.37	268	235	0.123	0.100
0.49	0	–	–	0.000	0.020
0.5*	0	–	–	0.000	0.000

*Simulations were not run for these limiting cases.

$\mu_{{n_{f} }}$: mean of $n_{f}$ from $N \times M$ = 500,000 simulations

$$\mu_{{n_{s} }}$$: mean number of crack segments going sideways (left or right) before reaching $$m$$$$m$$

As demonstrated by Table 1, the probability of failure computed numerically is very close to the theoretical value, $1 - 2q$. Therefore, the numerical simulations prove, again, the theory stated earlier. Moreover, from the table, it is clear that the number of crack segments leading to failure increases exponentially with $q$ until $q = 0.45$ after which it reaches a vertical asymptote when $q \to 0.5$, which means it is certain that cracks will take infinite time to bring the concrete surface to failure.

Note that in Table 1, for $q = 0.4$ 5, when the crack is left to propagate in any direction with at most $n_{{{\text{max}}}} =$ 300 walks, the total number of failures that is expected is only 37% out of 500,000 simulated cracks. However, if the crack is given enough time, all cracks will eventually lead to failure.

**Figure 18:**
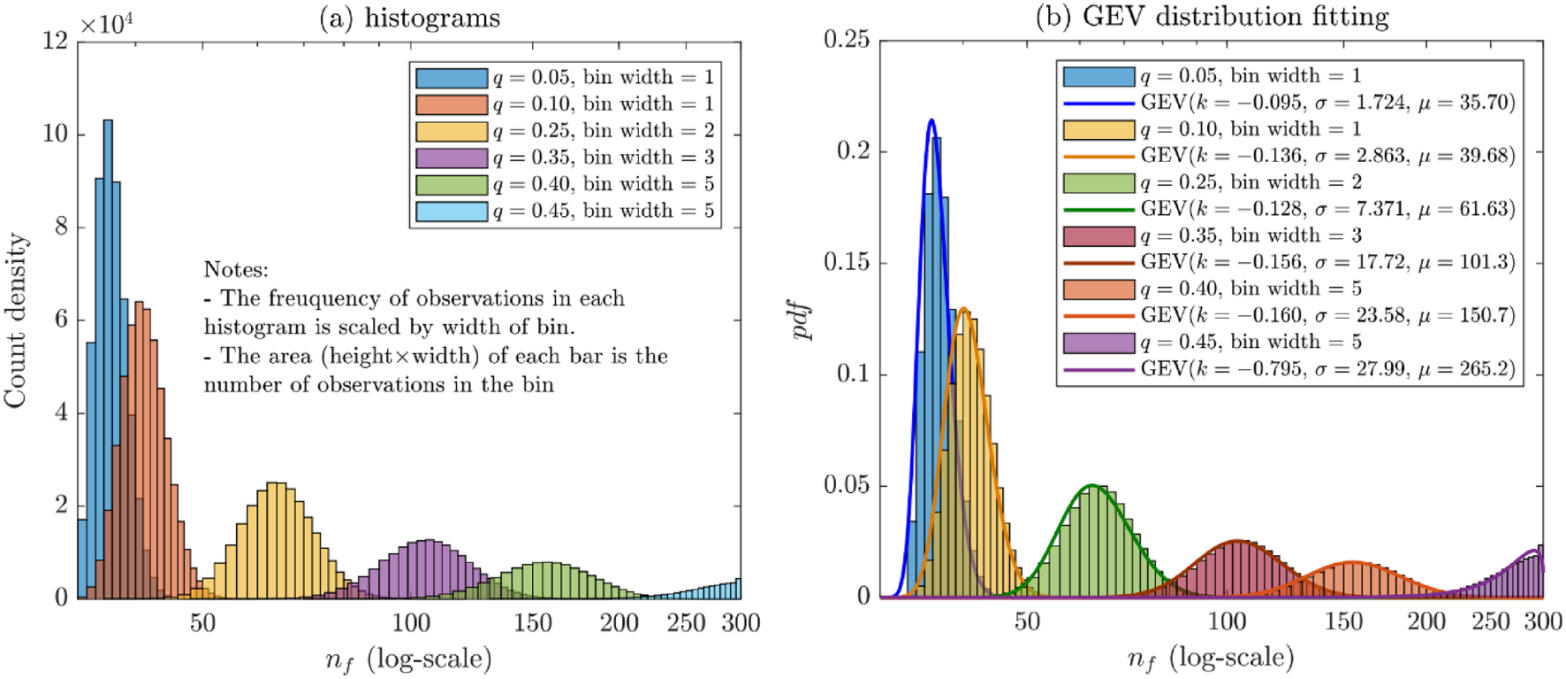
(**a**) $n_{f}$ histograms and (**b**) GEV distribution fitting for different values of the probability $q$.

Figure 18a shows the count density histograms of the number of crack segments leading to failure, $n_{f}$, for a number of probabilities $q$. The figure demonstrates important observations that were naturally expected. First, when $q$ is small (higher probability of moving forward), $n_{f}$ is small, and as $q$ increases, $n_{f}$ increases. The second important observation is that failure is almost certain when cracks are allowed to propagate more than 300 walks (or $> 300{\Delta }t$, assuming each crack segment takes $1{\Delta }t$, with ${\Delta }t$ is a unit of time) as long as $q \le 0.45$. In addition, the number of failures is correlated with $q$; given a short amount of time greater than 33 ${\Delta }t$, a small probability $q$ almost certainly leads to failure and vice versa.

The Generalized Extreme Value distribution (GEV) is found to be the best fit to all $q$ cases, as demonstrated in Fig. 18b. The GEV is often used in the modeling of the smallest or largest value in a large set of i.i.d random values of observations or measurements^29^. In the present subject, the observations are the values of $n_{f}$, representing the smallest number of walks (crack segments) leading to the failure of the concrete surface.

The GEV has three parameters: location, $\mu$, scale, $\sigma$, and shape, $k$. When $k < 0$, which is the case for all probabilities $q$ in the present simulations, the GEV is referred to as the Type III (Weibull) distribution.

Note that because the shape parameter $k < 0$, the distribution is right-truncated such that the probability density for $x < - \frac{\sigma }{k} + \mu$ is zero. This will have important implications in our subject matter. For instance, for $q = 0.05$, the GEV that fits the simulated data well has the following parameters (see Fig. 18b): $k = - 0.095$, $\sigma = 1.724$, and $\mu = 35.70$, which means that $P\left( {X > x = - \frac{\sigma }{k} + \mu } \right) = P\left( {X > x \approx 54} \right) = 0$. Therefore, according to the binomial distribution $P\left( {33;54{|}q = 0.05} \right) = 1$, failure is certain after 54 trials, i.e., when the pavement is left without maintenance for a continuous 54 ${\Delta }t$ time units after the formation of the crack. For the rest of the $q$ probabilities, the same formula can be used to estimate the limiting values of $n_{f}$, after which the probability of reaching failure is exactly one.

Furthermore, using the GEV parameters from Fig. 18b, we can compute the expected time to failure (ETTF) or the expected number of crack segments to failure. The ETTFs are found to be exactly the same as $\mu_{nf}$ values reported in Table 1 column #3. For example, for a crack that has a probability of propagating forward less than to the left and right, say $p = 1 - 2q = 0.2$, we expect the concrete pavement surface to fail after 161 crack segments or 161 ${\Delta }t$ time. The coefficient of variation in this case is about 16%.

## Concluding remarks

This article presents probabilistic models for the simulation of the propagation and orientation of concrete cracks in rigid concrete surface pavements. The models take into account the uncertainty in crack orientation. The results of these models can be used to estimate the probability of failure of concrete pavements, estimated time to failure, among other very important information that can be used in the decision making. All models are based on the assumption that the orientation of a crack is a random variable. The probabilities that a crack takes in a certain direction were computed using a sufficiently large database of real images of cracks in concrete pavements collected from the Mendeley Database.

The results of this study showed that the crack orientation is not uniformly distributed but is instead clustered around certain orientations following a normal distribution with an increasing coefficient of variation from the origin, which has been described by the diffusion phenomenon.

The probabilistic models were shown to accurately predict the distribution of crack orientations, probability of failure, and their potential use. The results of the model can be used to estimate the probability of failure of a concrete pavement and to identify the critical orientations of cracks, which are most likely to cause failure.

The probability of failure due to the propagation of cracks to a certain length can be used to design concrete surfaces in such a way to reduce it by increasing the probability of the cracks moving to the left and right. Such a design may involve the use of fibers (i.e., to make fiber reinforced concrete) in certain locations to increase $q$ and thus decrease $P_{f}$. The fibers come in different varieties, and their role is to bridge the gaps caused by cracks, thus orienting the cracks in a particular direction. Of course, this requires advanced numerical methods (such as nonlinear finite element analysis, methods based on discrete particle dynamics, etc.) or laboratory tests to achieve the optimum amount and location of fibers to be used when mixing the concrete and constructing the pavement surface.

Nevertheless, the developed models have some limitations. One limitation is that the models do not take into account the interaction between cracks.

## Data Availability

All data are available in the open source database of images and from within the manuscript.

## Software availability

The algorithm is entirely described in the manuscript. The GUI software, however, is not open source yet. The authors will decide on where to host-it for wider use by the community.
